# Effects of MRgFUS Treatment on Musculoskeletal Pain: Comparison between Bone Metastasis and Chronic Knee/Lumbar Osteoarthritis

**DOI:** 10.1155/2019/4867904

**Published:** 2019-09-02

**Authors:** Hirofumi Namba, Motohiro Kawasaki, Masashi Izumi, Takahiro Ushida, Ryuichi Takemasa, Masahiko Ikeuchi

**Affiliations:** ^1^Department of Orthopaedic Surgery, Kochi Medical School, Kochi University, Nankoku, Kochi 783-8505, Japan; ^2^Pain Medical Center, Shikoku Medical Center for Children and Adults, Zentsuji, Kagawa 765-8507, Japan; ^3^Multidisciplinary Pain Centre, School of Medicine, Aichi Medical University, Nagakute, Aichi 480-1195, Japan

## Abstract

Local bone denervation by magnetic resonance-guided focused ultrasound (MRgFUS) is a promising tool for alleviation of pain in patients with painful bone metastasis (BM). Considering the underlying mechanism of pain alleviation, MRgFUS might be effective for various bone and joint diseases associated with local tenderness. This study was conducted to clarify the therapeutic effect of focused ultrasound in patients with various painful bone and joint diseases that are associated with local tenderness. Ten patients with BM, 11 patients with lumbar facet joint osteoarthritis (L-OA), and 19 patients with knee osteoarthritis (K-OA) were included. MRgFUS treatment was applied to the bone surface with real-time temperature monitoring at the target sites. Pain intensity was assessed using a 100 mm numerical rating scale (NRS) at various time points. Pressure pain threshold (PPT) was evaluated on the sonication area and control sites. Compared to baseline, the pain NRS scores significantly decreased in all groups 1 month after treatment, and PPT at the treated sites significantly increased in all groups 3 months after treatment. The percentage of patients who showed *a* ≥ 50% decrease in pain NRS scores at 1 month after treatment was 80% in BM, 64% in L-OA, and 78% in K-OA groups. PPTs were significantly higher after treatment at all evaluation time points. This study indicated that MRgFUS is effective in reducing pressure pain at the site of most severe tenderness in patients with painful bone and joint diseases. Treatment response was comparable between patients with BM, L-OA, and K-OA.

## 1. Introduction

Magnetic resonance-guided focused ultrasound (MRgFUS) treatment is a noninvasive technique of localized thermal ablation by precisely focusing acoustic energy at the target site [1]. Recently, MRgFUS has been shown to have an antitumor effect, along with alleviating pain associated with bone metastasis (BM) [2, 3]. It has also been shown to alleviate chronic noncancer pain, such as that associated with lumbar facet joint osteoarthritis (L-OA) and knee osteoarthritis (K-OA) [4, 5].

The pain alleviation mechanism is believed to be due to heat-induced protein denaturation that is confined to the focal region. HIFU acts on hypersensitive nerve fibers and causes denaturation of increased neurotransmitters at the target site, leading to pain alleviation. During bone irradiation with focused ultrasound, most of the energy is absorbed by the bone surface; this increases the temperature of the bone surface and the contiguous soft tissues rather than the deeper bone tissues. Therefore, the pain mitigation effect is largely attributable to degeneration of nociceptors and primary afferent sensory nerve fibers on the bone surface. This hypothesis is reinforced by evidence that unmyelinated nerve fibers are particularly vulnerable to (non-HIFU) thermal injury [6], and that direct sonication of neuronal structures *in vivo* results in demyelination and neural degeneration [7].

Considering the pain alleviation mechanism resulting from local bone denervation, this treatment might be effective against pain associated with BM, as well as chronic joint pain due to osteoarthritis of the knee joint and lumbar facet joints. Based on the premise that HIFU treatment is likely to be effective against joint pain, if the bone surface is considered to be the major source of pain, we have pioneered the use of this method for treating chronic pain associated with medial K-OA.

Several studies, including randomized controlled trials, have demonstrated HIFU's effectiveness for pain associated with BM [3]; however, only few reports have described its effectiveness in patients with other bone and joint diseases [4, 5, 8]. In addition, HIFU treatment is not effective for all patients with painful BM. Therefore, it is unclear which types of cases respond to treatment. Moreover, although nerve degeneration at the bone surface is believed to be the mechanism of pain relief, there is no clinical report such as a report showing local necrosis after the HIFU treatment for BM in a pig model [9]. There is also no research quantitatively evaluating local pain at the irradiation site, other than images and pathological evaluations, except for the posttreatment measurement of pressure pain threshold (PPT) at the site of pain conducted at our facility [5]. To evaluate the therapeutic effect of MRgFUS in patients with tenderness at the target sites, we focused on the importance of change in the PPT, as well as change in the patients' subjective sense of pain, in this study.

So far, our experience with MRgFUS for BM, L-OA, and K-OA has indicated that it has high therapeutic value, although it has also raised the question of the most effective positioning of the therapy. In this regard, we hypothesize that this therapy is most effective when applied at the point of maximal tenderness.

The purpose of this study was to elucidate the changes in PPT and pain induced by applying this therapy to the region of maximal tenderness in patients with three types of bone diseases.

## 2. Patients and Methods

### 2.1. Patients

At our facility, since 2008, three clinical studies have been conducted to evaluate pain relief with the use of MRgFUS in patients with BM, L-OA, and K-OA. The subjects of the current study were retrospectively extracted from these three studies. We enrolled patients with intractable pain (numerical rating scale (NRS) score ≥4) that persisted for more than 3 months and with bone surface tenderness. Only patients who would be available for clinical evaluation at each evaluation time were included.

As a result, ten patients with painful BM, 11 patients with chronic low back pain associated with L-OA, and 18 patients with chronic medial knee pain associated with K-OA were included in this study.

The exclusion criteria for the three clinical trials were patients on dialysis, unstable cardiac status, severe hypertension, active uncontrolled diseases such as infection, hematological diseases, neurological diseases, severe cerebrovascular diseases, severe coagulation disorders, or use of anticoagulant/platelet drugs, mental handicaps, or psychiatric conditions that precluded adequate communication, contraindications for MR imaging due to implanted metallic devices, claustrophobia, or obesity (weight >113 kg).

The inclusion criteria for BM patients were pain refractory to conventional palliative therapy, such as radiation and/or opioids, age >18 years, number of metastatic bony lesions <3, tumor diameter <8 cm, tumors located at least 1 cm away from the skin, internal organs and nerves, low risk of pathological fractures (Mirels' score ≤7) [10], life expectancy >6 months, and ability to carry on normal activities (Karnofsky Performance Scale (KPS) score >70) [11].

The inclusion criteria for patients with L-OA were chronic low back pain refractory to other conservative treatments for >6 months, age >65 years, number of targeted facet joints with tenderness  <2, at least 70% pain reduction achieved with diagnostic facet joint block using 1% lidocaine, and absence of radicular pain.

The inclusion criteria for patients with K-OA were chronic medial knee pain refractory to other conservative treatments for >6 months, surface of the target bone at least 1 cm distant from skin, age >60 years, no severe instability of the knee joint with tenderness, and grade 3 or 4 medial knee OA according to the Kellgren–Lawrence classification [12].

The treatment sites in patients with BM were the ilium (4 patients), sacrum (2 patients), femur (2 patients), scapula (1 patient), and pubic bone (1 patient). The sources of metastasis were prostate cancer (2 patients), myeloma (2 patients), and one case each of hepatoma, uterine cancer, lung cancer, thyroid cancer, breast cancer, and adenoid cystic carcinoma. On radiological examination of patients with BM, four patients had osteolytic lesions and the remaining had mixed and/or osteoblastic lesions, but the structure of bone surface in the treatment area of all patients was almost maintained.

### 2.2. Methods

MRgFUS was applied to the most tender region over the metastatic tumor in the BM group, the dorsal area of the painful facet joints, which was selected based on 70% reduction of pain with the diagnostic block in the L-OA group, and the bone surface around osteophytes in the medial tibiofemoral joint in the K-OA group. Before FUS treatment, the points of tenderness around the painful area were meticulously identified. Once the most tender point was identified, the planned focal FUS treatment was to be applied exclusively at that point and the surrounding area.

### 2.3. MRgFUS

All procedures were performed under MRI (GE Sigma EXCITE 3.0 T MRI, Milwaukee, WI, USA) guidance. Two FUS systems were used: the ExAblate® 2000 system (InSightec Ltd., Haifa, Israel), integrated with the MRI table, was used in patients with BM and L-OA. With the ExAblate® 2000 system, the patient was made to lie recumbent with the lesion in contact with the FUS transducer embedded in the MRI table, a gel pad was placed between the transducer and the lesion, and the periphery was filled with degassed water. The ExAblate® 2100 conformal bone system (InSightec Ltd., Haifa, Israel) was used for K-OA patients. A ball filled with degassed water was formed on the irradiated side of the transducer in this system, and the transducer was placed in contact with the body surface and secured with a strap.

The target area was determined in accordance with the disease group. For BM, the target site was the surface of bone with maximum tenderness among the areas of BM, as detected in advance by the physical examination; for K-OA, it was the bone surface at the region of maximum tenderness on the inner side of the tibiofemoral joint; for L-OA, it was the bone surface on the dorsal side of the facet joint identified in advance, where transient relief from tenderness was obtained by the diagnostic nerve block.

The transducer was placed on the body with the selected target area at its center, and before initiation of treatment, it was confirmed by MRI that irradiation could be performed without a problem. The location of the transducer was also confirmed on the MRI monitor before treatment. If the location was found unsuitable, the procedure for confirmation of the suitable transducer position by MRI was repeated, while changing the placement position.

After determining the target area, several parameters, including the required energy level, number of focused ultrasound energy deliveries (sonications), and the direction of the pathway of the beam, were automatically optimized.

**Figure 1:**
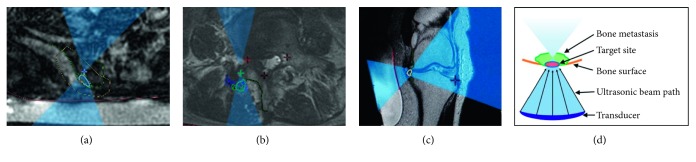
The pictures show the corresponding ultrasonic irradiation pathways on the MRI monitor. The blue area represents the ultrasonic irradiation route, and the green circle is the target site for each disease. (a) Irradiation for painful bone metastasis of the ilium. The green circle shows the part with maximal tenderness over the bone metastasis site in the ilium. (b) Irradiation for lumbar facet joint osteoarthritis. The green circle shows the dorsal area of the painful lumbar facet joint. (c) Irradiation for knee osteoarthritis. The green circle shows the bone surface around osteophytes in the medial tibiofemoral joint. (d) Schematic diagram of the pathway of the ultrasonic irradiation to the bony lesion.

Actual views of the monitor screen when setting the irradiation path of ultrasonic waves for BM, K-OA, and L-OA are shown in Figures 1(a)–1(c), respectively, and the ultrasonic irradiation pathway to the bone lesion is shown schematically in Figure 1(d).

A sonication session lasted approximately 20 seconds and was repeated after a cooling period of >90 seconds between each sonication. Initially, low energy sonication was applied as a test to ensure safety and accuracy. Then, therapeutic sonication begun with a higher energy level was used to complete ablation.

Throughout the treatment, the location of each sonication and the temperature of the tissues adjacent to the target area were monitored in real time, using proton resonance frequency shift thermometry [13]. The target therapeutic temperatures at the treatment sites were 60°C in the BM group and 55°C in L-OA and K-OA groups. The target temperature was attained by manually raising the irradiation energy and narrowing and adjusting the irradiated region. The actual mean sonication energies for BM, L-OA, and K-OA in this investigation were 976 J, 458 J, and 724 J, respectively. The patients were allowed to interrupt treatment at any time by pushing the stop button.

### 2.4. Clinical Evaluation

#### 2.4.1. Pain Numerical Rating Scale (NRS) Score

The primary outcome measure was the worst pain at the painful area indicated by NRS scores in the last 24 hours (range: 0 (no pain) to 10 cm (worst pain)). A response to treatment was defined as *a* ≥ 50% decrease in NRS scores, as recommended by the Outcome Measures in Rheumatology Clinical Trials and Osteoarthritis Research Society International (OMERACT-OARSI) [14]. NRS scores were assessed before treatment and at 1 week, 1 month, and 3 months after treatment.

#### 2.4.2. Pressure Pain Threshold (PPT)

PPT was defined as the first instance at which the patients perceived the pressure as slight pain. We assessed the PPT using an electronic pressure algometer at the most tender point. PPT was evaluated at the treatment site and at the corresponding contralateral, nontreated site as a control. For the measurement, the area of maximum tenderness was identified before treatment and marked as the target treatment site. Then, the direction and distance of this site from an anatomical landmark was recorded by photography, to avoid region variation during subsequent measurements. The measurement was thus performed at the same point in sequential assessments at all posttreatment time points.

A handheld algometer (Algomed, Medoc Ltd., Ramat Yishai, Israel) with a 1 cm^2^ probe was used to evaluate PPTs. The measurements of PPTs were performed by two examiners (MK or HN) before treatment and at 1 week, 1 month, and 3 months after treatment. PPTs were measured four times at each evaluation point, and the average of the remaining two values, excluding the maximum and minimum values, was used for analysis.

### 2.5. Statistical Analysis

Unpaired *t* test, chi-squared test, and Fisher's exact test were used to compare demographic data and treatment responders. Pain NRS scores and PPT data are presented as the median (interquartile range). Friedman test, followed by Dunn's post hoc test, was used to evaluate the time course of pain NRS scores and PPT in each group. Friedman test, followed by Dunn's post hoc test, was used to evaluate the time course of PPT at every assessment point. Mann–Whitney's *U* test was used to compare the PPT between responders and nonresponders at each assessment point. A significant difference was set at *p* < 0.05. Statistical analysis was conducted with GraphPad Prism 7 software (GraphPad Software Inc., San Diego, CA) for Windows (Microsoft Corporation, Redmond, WA, USA).

## 3. Results

In the BM group, 10 patients completed the treatment, while one patient withdrew due to anxiety. Assessment of pain NRS scores and PPT was completed in 10 and 8 patients, respectively. In the L-OA group, all patients completed the treatment. Assessment of pain NRS score and PPT was completed in 11 and 8 patients, respectively.

In the K-OA group, 18 patients completed the treatment, while one patient could not complete the planned sonication treatment because of anxiety during the treatment. Assessment of pain NRS scores and PPT was completed in 14 and 9 patients, respectively. Total knee arthroplasty was subsequently performed in four patients who expressed a desire for the procedure because pain was not sufficiently alleviated within the first 3 months posttreatment or was more severe than expected. The patients' baseline characteristics are shown in Table 1.

**Figure 2:**
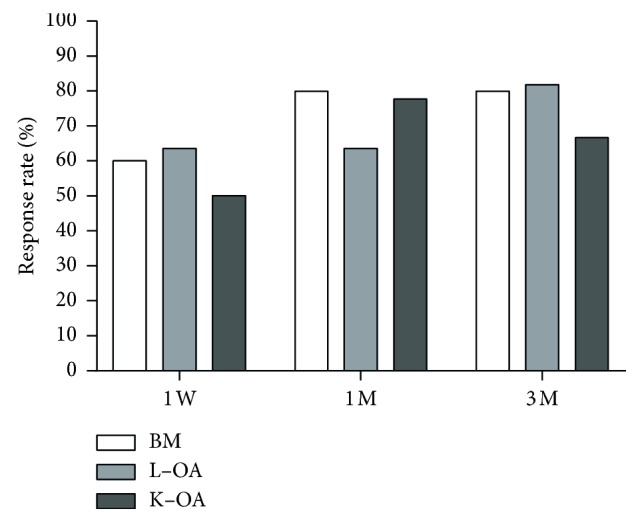
Percentage of patients who responded to treatment at various time points (defined as *a* > 50% decrease in the pain NRS score). NRS, numerical rating scale; BM, bone metastasis; L-OA, lumbar facet joint osteoarthritis; K-OA, knee osteoarthritis; W, week; M, month.

In the BM group, the percentage of patients who responded to FUS treatment was 60% at one week, 80% at 1 month, and 80% at 3 months after treatment. The corresponding percentages in the L-OA group were 64% at one week, 64% at 1 month, and 82% at 3 months, while those in the K-OA group were 50% at one week, 78% at 1 month, and 67% at 3 months (Figure 2). Although the response rates at 1 month in the BM and K-OA groups were higher than those in the L-OA group and those in the BM and L-OA groups at 3 months were higher than those in the K-OA group, the differences were not significant.

**Figure 3:**
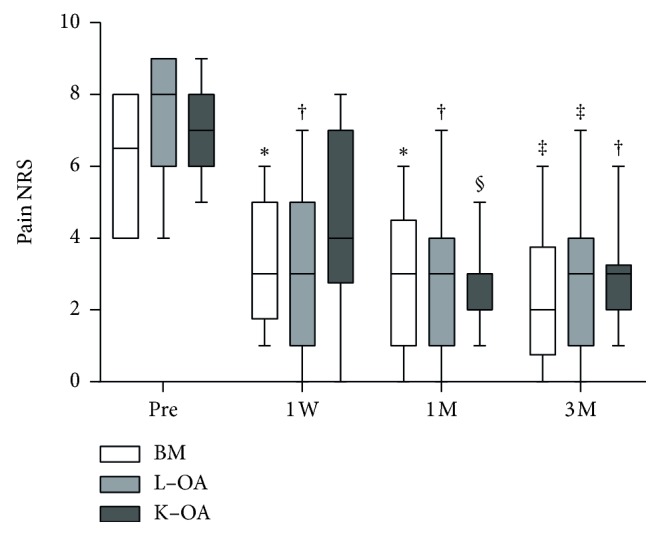
Pain NRS scores of patients at various time points. White, light gray, and gray bars indicate pain NRS scores of patients with BM (*n* = 10), L-OA (*n* = 11), and K-OA (*n* = 18), respectively. ^*∗*^*p* < 0.05 versus pretreatment scores; ^†^*p* < 0.005 versus pretreatment scores; ^‡^*p* < 0.0005 versus pretreatment scores; ^§^*p* < 0.0001 versus pretreatment scores. BM, bone metastasis; L-OA, lumbar facet joint osteoarthritis; K-OA, knee osteoarthritis; NRS, numerical rating scale; Pre, pretreatment; W, week; M, month.

**Figure 4:**
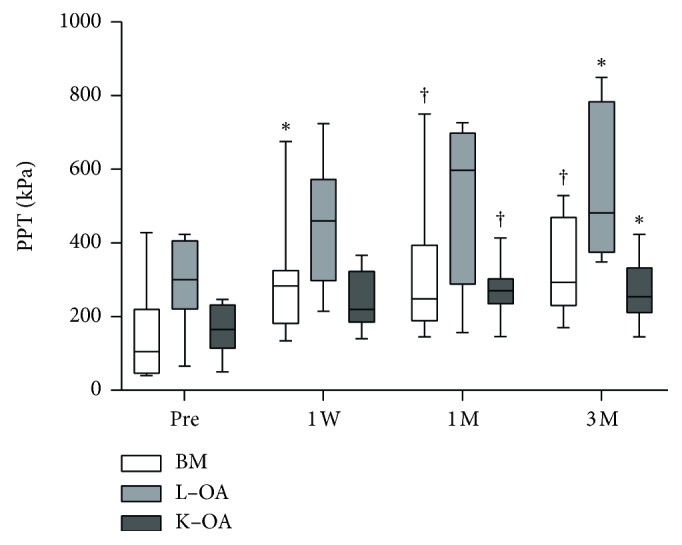
Pressure pain thresholds of patients at various time points. White, light gray, and gray bars indicate the PPT of patients with BM (*n* = 8), L-OA (*n* = 8), and K-OA (*n* = 9), respectively. ^*∗*^*p* < 0.05 versus pretreatment PPT; ^†^*p* < 0.005 versus pretreatment PPT. PPT, pressure pain threshold; BM, bone metastasis; L-OA, lumbar facet joint osteoarthritis; K-OA, knee osteoarthritis; Pre, pretreatment; W, week; M, month.

**Figure 5:**
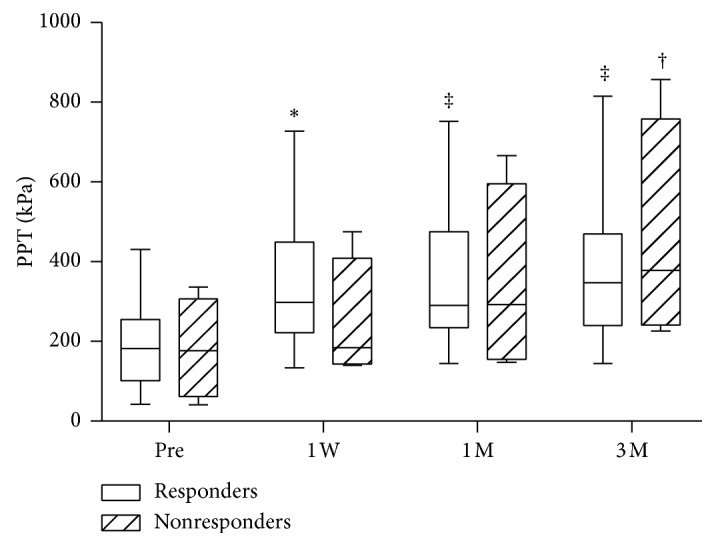
Pressure pain thresholds of responders and nonresponders at various time points. White and shaded bars indicate the PPT of responders (*n* = 21) and nonresponders (*n* = 4), respectively. ^*∗*^*p* < 0.01 versus pretreatment PPT; ^†^*p* < 0.001 versus pretreatment PPT; ^‡^*p* < 0.0001 versus pretreatment PPT. PPT, pressure pain threshold; Pre, pretreatment; W, week; M, month.

The median NRS score (min–max) significantly decreased from 6 (4–8) at baseline to 2 (0–6) at the final follow-up in the BM group (*p* < 0.0005), from 8 (4–9) to 3 (0–7) in the L-OA group (*p* < 0.0005), and from 7.5 (5–9) to 3 (1–9) in the K-OA group (*p* < 0.005); however, there were no significant differences between the three groups with respect to pain reduction (Figure 3). The median PPT (min–max) at the treatment sites significantly increased from 107 kPa (40–432) at baseline to 271 kPa (94–534) at the final follow-up in the BM group (*p* < 0.005), from 273 kPa (66–427) to 487 kPa (352–858) in the L-OA group (*p* < 0.05), and from 156 kPa (50–249) to 246 kPa (120–427) in the K-OA group (*p* < 0.005) (Figure 4). There were no significant differences between pre- and posttreatment PPT at the control sites in all three groups. The median posttreatment PPT in responders was significantly higher at all evaluation time points compared to pretreatment PPT. However, a significant increase in PPT was observed only three months in nonresponders (Figure 5).

## 4. Discussion

Although previous studies have shown consistently good results of MRgFUS treatment for pain management in BM patients [3, 15, 16], only few studies have investigated the efficacy of MRgFUS against the pain of L-OA and K-OA [4, 5]. Furthermore, no studies have compared the therapeutic effects of MRgFUS in patients with different diseases, and it is not clear which painful diseases are suitable targets for MRgFUS treatment. Therefore, we compared the effects of MRgFUS treatment in patients with three diseases. Although we hypothesized that the pain relieving effects of MRgFUS for L/K-OA would be inferior to those for BM, we observed similar efficacy in patients with L/K-OA.

Since BM is a progressive condition, the associated pain is liable to recur due to disease progression. However, in the present study, patients with BM showed significantly reduced pain for more than 3 months after treatment. There are several factors that could explain the efficacy of FUS against the pain associated with BM. First, the treatment causes denaturation of not only the sensory nerves but also the superficial part of the lesion itself. Therefore, the treatment might prevent lesion expansion to some extent. Second, pain associated with BM is not usually generated by joint motion, in contrast to that with L/K-OA. Lastly, a large proportion of patients did not have rapidly progressing tumors.

L/K-OA is a chronic rather than rapidly progressive disease. The pain associated with L/K-OA usually correlates with mechanical stress. Since abnormal mechanical stress cannot be eliminated by MRgFUS treatment, pain associated with motion is liable to recur. Therefore, we hypothesized that the pain associated with L/K-OA was more likely to recur than that due to BM. However, the effect of pain relief was almost the same in all three groups in this study. There are several possible reasons for the good pain relief effect in patients with OA. First, although the pain associated with OA is caused by various mechanisms, patients with obvious localized pain at the medial knee was selected in this study. Second, patients who had severe knee and lumbar instability were excluded from the study. Lastly, denervation of sensory nerves without any damage to the joints aborts the nociceptive input without necessarily affecting the disease course.

Many researchers have reported the pain relieving effect of MRgFUS on painful BM [15, 16]. In a recent multicenter, randomized, placebo-controlled trial of MRgFUS treatment for BM [3], 72 out of 112 patients (64.3%) were responders in the MRgFUS arm at 3 months, compared to 7 out of 35 (20.0%) patients in the placebo arm, as assessed using the international consensus on palliative radiotherapy endpoints for future clinical trials in bone metastasis [17]. The response rate in our study was higher than that reported in previous studies. This might be attributable to study population characteristics. Furthermore, since the pain in patients with BM involves various factors, in the present study, we selected patients with tenderness and applied the focused ultrasound wave irradiation at the most tender point of the targeted BM. Thus, the patients selected were those who presented hypersensitive pain on pressure stimulation of the body surface. Moreover, the higher response rate could also be the result of prioritized treatment in the surface region that was hypersensitive to physical stimulation due to BM.

Only one previous study by Weeks et al. has investigated the use of MRgFUS treatment in L-OA patients [4]. In their study, 12 out of 18 (67%) patients showed reduction in worst NRS pain scores, and the percent reduction in the pain NRS score was 51.2%. We believe that if the NRS pain scores of these 12 patients were judged based on the OMERACT-OARSI criteria, all 12 patients would have been considered to be responders, and the response rate of their research would have been equivalent to our results.

Furthermore, the average number of facet joints treated in their study was 4.9. In contrast, the corresponding number in our study was 1.2. Despite fewer treatment sites in our study, the therapeutic effects in both studies were equivalent. This is likely attributable to the selection of only patients who had tender points on their backs in our study. Therefore, the treatment was effective owing to the appropriate identification and treatment at the points of maximal tenderness.

Only one previous study, conducted by Izumi et al. at our institution, has investigated MRgFUS treatment in K-OA patients [5]. In our study, six out of eight (75%) patients were categorized as responders according to the OMERACT-OARSI criteria. The inclusion criteria in the previous preliminary study and the response rate were equivalent to those of our current study.

At our facility, we have treated patients with BM, L-OA, and K-OA who have tenderness. In the present study, comparing the effects in these three disease groups, we found similar therapeutic effects for all three diseases. Moreover, in the group with treatment response, PPT began to increase significantly from an early stage. In other words, in both BM and OA, focal ablation of local hypersensitive nerve fibers seems capable of effectively reducing the level of pain (NRS score) experienced by the patient.

This is the first report in which the effects of MRgFUS treatment were investigated using tender sites in bone and joint diseases with various manifestations of pain. This study shows that the efficacy of the treatment in osteoarthritis is similar to that in painful BM. In addition, the response to treatment was higher than that in previous studies conducted in patients with BM and L-OA. We believe that this is attributable to meticulous detection and targeting of the most painful sites in our study. For MRgFUS treatment, it is important to select patients with tender points in the painful areas.

The results in the three groups in our study shows that meticulous detection of the point with the lowest PPT as the main treatment area is likely to permit identification of the eligible lesion, thus optimizing the results.

In the current study, we have only reported on the short-term results of MRgFUS, which is a limitation of the study. Future studies should evaluate the long-term outcomes of treatment. However, our study demonstrates the potential of FUS as a promising treatment for degenerative musculoskeletal diseases. Some other limitations should also be acknowledged, the most important of which is that this was a small case-series without a control group. Hence, it is difficult to rule out a placebo effect. Secondly, two types of treatment devices (ExAblate 2000 and 2100) were used in this study, which might have affected the degree of pain relief. However, we believe that this was of little consequence as temperature elevation was confirmed by MRI monitoring during treatment.

## 5. Conclusions

This study showed that MRgFUS reduced pain when applied at the site of most severe tenderness in patients with painful bone and joint diseases. The treatment response was comparable in patients with BM, L-OA, and K-OA. MRgFUS increased the threshold of tenderness at the lesion site in all three disease groups.

## Figures and Tables

**Table 1:** Baseline characteristics of patients in the three study groups.

	BM	L-OA	K-OA
Number	10	11	18
Age (years)	69 (41–80)	74 (64–85)	80 (60–83)^*∗*^
Female	4	8	14
Pain NRS score	6 (4–8)	8 (4–9)	7.5 (5–9)

Patients with K-OA were significantly older than patients with BM and L-OA. Data are presented as median (min–max); ^*∗*^*p* < 0.05 versus BM and L-OA. BM, bone metastasis; L-OA, lumbar facet joint osteoarthritis; K-OA, knee osteoarthritis; NRS, numerical rating scale.

## Data Availability

The data used to support the findings of this study are available from the corresponding author upon request.
